# In-Depth Biological Activities, Chemical Profiling by LC-MS/MS of Mersin, Türkiye Bee Products (Pollen, Honey, Propolis, Royal Jelly and Bee Bread)

**DOI:** 10.3390/molecules31183246

**Published:** 2026-09-14

**Authors:** Ebubekir İzol, Mustafa Abdullah Yılmaz, İlhami Gülçin

**Affiliations:** 1 Bee and Natural Products R&D and P&D Application and Research Center, Bingol University, 12000 Bingöl, Türkiye; 2 Department of Bee and Bee Products, Institute of Science, Bingöl University, 12000 Bingöl, Türkiye; 3 Department of Pharmaceutical Chemistry, Faculty of Pharmacy, Dicle University, 21280 Diyarbakır, Türkiye; mustafaabdullahyilmaz@gmail.com; 4 Ağrı İbrahim Çeçen University, 04100 Ağrı, Türkiye; 5 Department of Chemistry, Faculty of Sciences, Atatürk University, 25240 Erzurum, Türkiye

**Keywords:** bee products, pollen, antioxidant, biological activity, chemical ingredient, LC-MS/MS

## Abstract

Bee products, including propolis, royal jelly, honey, bee pollen, and bee bread, are natural functional foods recognized for their nutritional value and health-promoting properties. In the present study, propolis, royal jelly, honey, bee pollen, and bee bread collected from Mersin, one of Türkiye’s major beekeeping regions, were comprehensively characterized in terms of their phytochemical composition, antioxidant potential, enzyme inhibition effects, and physicochemical properties. The antioxidant capacities of the samples were evaluated by determining total phenolic content (TPC), total flavonoid content (TFC), and their radical scavenging and reducing abilities using the DPPH, ABTS, and DMPD radical scavenging assays and CUPRAC, FRAP, and Fe^3+^-reducing assays. Enzyme inhibition effects were assessed against human carbonic anhydrase I and II (hCA I and hCA II), acetylcholinesterase (AChE), butyrylcholinesterase (BChE), α-glycosidase, and α-amylase. Furthermore, the phytochemical profiles of all bee products were determined by LC–MS/MS through the targeted analysis of 53 phenolic compounds and related metabolites. Furthermore, the honey sample was evaluated for its principal physicochemical quality parameters, including hydroxymethylfurfural (HMF), sugar composition (glucose, fructose, sucrose, and maltose), naphthalene residue, diastase activity, proline content, free acidity, pH, moisture content, and electrical conductivity. Consistent with its phytochemical composition, propolis showed the strongest antioxidant activity among the investigated bee products under the same experimental conditions. These findings highlight the antioxidant potential of Mersin bee products and indicate their potential as natural sources of bioactive compounds, with possible applications in functional food and nutraceutical research. Further comprehensive studies are warranted to better characterize their bioactive constituents and explore their potential applications.

## 1. Introduction

The Mediterranean region of Türkiye, including Mersin, has a rich and diverse flora with a high proportion of endemic plant species (3321 endemic localities, 34.3%). Its favorable climate also enables long-term beekeeping activities and attracts numerous migratory beekeepers. These distinctive botanical and climatic characteristics may influence the chemical composition and biological properties of bee products [1]. However, Mersin bee products have not yet been comprehensively investigated in terms of their phytochemical, antioxidant, enzyme inhibitory, and physicochemical properties, highlighting the relevance of the present study.

Bee products represent an important group of natural products that have been used for centuries for nutritional, traditional, and health-related purposes. Honey is the most widely recognized and consumed bee product; however, beekeeping also provides a diverse range of other products, including bee pollen, propolis, royal jelly, bee bread (perga), bee venom, beeswax, and bee larvae, each of which possesses distinct nutritional and chemical characteristics and has attracted increasing scientific interest [2,3,4,5,6,7,8]. These products contain a wide variety of bioactive constituents, including polyphenols (including flavonoids), proteins, peptides, fatty acids, amino acids, vitamins, minerals, and other phytochemicals, which may contribute to their diverse biological properties Pollen constitutes an important nutritional and protein source for honey bees, while propolis, bee bread, and royal jelly have received considerable scientific attention because of their complex chemical composition and reported antioxidant, antimicrobial, anti-inflammatory, and other biological activities [9,10,11,12,13,14,15]. More broadly, research on bee products has demonstrated that their nutritional value and biological activities are closely associated with their diverse chemical profiles, which can vary according to botanical and geographical origin, environmental conditions, bee species, and processing or storage conditions. Therefore, bee products are increasingly being investigated as valuable sources of naturally occurring bioactive compounds with potential applications in food, nutraceutical, pharmaceutical, and health-related fields [16]. 

The simultaneous evaluation of antioxidant and enzyme inhibitory properties is an important approach for assessing the biological activities of natural products and elucidating their potential therapeutic and functional value [17]. Oxidative stress arises from an imbalance between the generation of reactive oxygen species and antioxidant defense systems and may lead to oxidative damage to cellular macromolecules. Therefore, identifying natural antioxidants capable of neutralizing free radicals and limiting oxidative damage is of great importance for the prevention of various chronic and metabolic diseases [18,19].

However, the biological potential of natural compounds should not be limited to their antioxidant properties, and their effects on different therapeutic targets should also be investigated. In particular, inhibition of acetylcholinesterase (AChE) and butyrylcholinesterase (BChE) is important in Alzheimer’s disease (AD) and other neurodegenerative disorders, as it may contribute to the preservation of cholinergic neurotransmission by reducing the degradation of acetylcholine [20]. Similarly, carbonic anhydrase (CA) I and II isoenzymes are important enzymatic targets due to their roles in acid–base homeostasis and various metabolic processes, and the inhibitory effects of natural polyphenolic compounds on these enzymes have attracted considerable biological interest [16].

On the other hand, inhibition of α-amylase and α-glycosidase can contribute to the control of postprandial glycemia by slowing the breakdown of complex carbohydrates and the release of glucose and is therefore considered an important strategy in the investigation of naturally derived antidiabetic agents [21]. Accordingly, the simultaneous assessment of the antioxidant properties of natural compounds and their inhibitory effects on cholinesterases, carbonic anhydrase, and carbohydrate-digesting enzymes enables a comprehensive evaluation of their antioxidant, neuroprotective, and antidiabetic potential rather than focusing on a single biological activity. Therefore, investigating the enzyme inhibition profiles and antioxidant capacities of natural sources provides an important scientific approach for identifying biologically active compounds and supporting the development of novel functional and therapeutic agents.

Honey is an important bee product that has traditionally been valued for its health benefits in addition to its nutritional properties. However, owing to its high economic value, adulteration and fraudulent practices in honey represent significant food safety and consumer health concerns. Therefore, the assessment of analytical parameters for determining honey quality and authenticity is of great importance. The hydroxymethylfurfural (HMF) level is an important indicator used to assess whether honey has been exposed to high temperatures or subjected to heat treatment [22]. The sugar profile provides valuable information, particularly for detecting the addition of synthetic sugars or sugar syrups to honey [23]. Proline and diastase values are among the key parameters used to evaluate honey quality and maturity [24]. 

In addition, the determination of undesirable residues such as naphthalene is important for identifying food safety risks that may arise from beekeeping practices [25]. Moisture content reflects the water content of honey and, consequently, its quality and storage stability, with lower moisture levels generally being associated with more favorable quality characteristics [26]. Electrical conductivity is a useful parameter for assessing the mineral content and concentration of honey, determining its botanical origin, and distinguishing blossom honey from honeydew honey [27]. Free acidity is another important quality criterion that provides information about the sensory characteristics and origin of honey. In particular, the levels of gluconic and citric acids may contribute to the evaluation of the floral or honeydew origin of honey [28].

In this study, the chemical composition, biological activities, and quality characteristics of five different bee products from the Mersin region of Türkiye were comprehensively investigated. In addition to their phytochemical profiles, the total phenolic and flavonoid contents were determined, antioxidant capacities were evaluated using six different assays, and inhibitory effects against six enzymes associated with metabolic and neurological disorders were assessed. Furthermore, the major quality characteristics of the bee products were comparatively examined. LC-MS/MS was employed for the identification of phytochemical constituents, HS-GC-MS for naphthalene analysis, HPLC-RID for characterization of the sugar profile, and HPLC-DAD for determination of HMF levels. Through this comprehensive analytical approach, the chemical profiles, antioxidant properties, metabolic enzyme inhibitory potentials, and quality indicators of Mersin bee products were evaluated collectively for the first time. The findings provide important scientific evidence regarding the biological, functional, and quality potential of these valuable natural products.

## 2. Results and Discussions

### 2.1. Chemical Content by LC-MS/MS

The quantitative profiles of 53 phytochemicals identified in bee products collected from Mersin Province, Türkiye, were determined by LC-MS/MS and are presented in Table 1. The analyzed compounds included a broad range of phenolic acids, flavonoids, and other bioactive phytochemicals, providing a comprehensive overview of the chemical composition of the investigated bee products. The concentrations of individual compounds varied considerably among the samples, indicating differences in their phytochemical profiles and suggesting that the botanical origin of the products may have a substantial influence on their chemical composition. Collectively, these findings provide valuable information on the phytochemical composition of bee products originating from Mersin Province and highlight the potential of these products as important natural sources of biologically active compounds.

The LC-MS/MS analysis revealed a diverse and product-specific chemical profile among the bee products obtained from Mersin Province. Propolis showed the richest profile and contained relatively high concentrations of several important phenolic acids and flavonoids, particularly acacetin (59.381 mg/g extract), naringenin (8.244 mg/g), chrysin (7.491 mg/g), ferulic acid (4.594 mg/g), apigenin (3.254 mg/g), and kaempferol (3.121 mg/g). Honey was characterized by a remarkably high concentration of salicylic acid (43.132 mg/g extract), followed by acacetin (5.751 mg/g), quercetin (3.068 mg/g), naringenin (2.769 mg/g), and chrysin (1.980 mg/g). These compounds are widely recognized for their antioxidant and other biological activities and may contribute to the functional properties of bee products.

Pollen and bee bread generally contained lower concentrations than propolis and honey but still showed a varied composition, including quercetin, isoquercitrin, astragalin, luteolin, and kaempferol. Royal jelly differed from the other products, with quinic acid (19.242 mg/g extract) and 4-OH benzoic acid (4.863 mg/g) being the most prominent compounds. Overall, the substantial differences in phytochemical composition among the products indicate that both product type and botanical origin may strongly influence their secondary metabolite profiles. The presence of numerous phenolic acids and flavonoids further supports the potential of Mersin bee products as valuable sources of bioactive compounds, although broader sampling and botanical characterization would be necessary to establish more definitive relationships between floral origin and phytochemical composition.

These results further demonstrate that the chemical composition of bee products obtained from regions with different geographical, climatic, and vegetation characteristics can vary considerably. In a study conducted in Morocco, polyphenolic compounds in honey, pollen, and propolis were determined. The highest concentrations were reported for caffeic acid in honey (34 mg/kg), pinocembrin in propolis (29.01 mg/kg), and caffeic acid in pollen (41 mg/kg) [29]. In the present study, caffeic acid was detected at concentrations of 2.87 mg/g in Mersin honey and 3.095 mg/g in propolis, whereas it was not detected in pollen. In another study, caffeic acid was reported as the most abundant polyphenol among the compounds investigated in royal jelly, while luteolin was detected at the lowest level [30]. In Mersin royal jelly, caffeic acid was determined at 0.077 mg/g, whereas luteolin was detected at a concentration of 0.002 mg/g. These findings further confirm that bee products from different geographical regions may exhibit distinct phytochemical profiles and substantial differences in the concentrations of individual compounds. In Mersin honey, the highest concentrations were observed for salicylic acid (43.132 mg/g), acacetin (5.751 mg/g), quercetin (3.068 mg/g), caffeic acid (2.87 mg/g), and naringenin (2.769 mg/g). The chemical structures of the phytochemicals detected at relatively high concentrations in the investigated bee products are presented in Table 2.

The biological relevance of the major phytochemicals detected in the investigated bee products was evaluated based on their previously reported activities. Among the compounds identified, several phenolic acids and flavonoids are known to possess a broad spectrum of biological properties. For example, caffeic acid has been widely investigated for its antioxidant, antitumor, antifibrotic, antihypertensive, and antiviral activities [31,32,33]. Similarly, *p*-coumaric acid has been associated with antioxidant, anticancer, antiviral, antimicrobial, antidiabetic, antihyperlipidemic, and anti-inflammatory effects [34,35,36]. The occurrence of these phenolic acids in Mersin bee products may therefore contribute to their overall biological potential.

Flavonoids identified in the samples also have considerable biological importance. Naringenin has been reported to exhibit antioxidant, antibacterial, anticancer, antiviral, and antifungal properties [37,38], whereas kaempferol has demonstrated antioxidant, anticancer, anti-inflammatory, antidiabetic, antiallergic, and antimicrobial activities [39,40,41]. Acacetin (5,7-dihydroxy-4′-methoxyflavone), another flavone derivative detected in the samples, has attracted considerable interest because of its reported anti-inflammatory and anticancer effects. In addition, Acacetin may provide useful chemotaxonomic information on the botanical origin of bee products, as its occurrence has been associated with *Robinia pseudoacacia* pollen. Therefore, its detection may provide an additional indication of the contribution of *R. pseudoacacia* as a floral source [42,43]. Chrysin (5,7-dihydroxyflavone) has likewise been associated with antioxidant, anti-inflammatory, anticancer, and antiviral activities [44,45]. Apigenin is another biologically relevant flavonoid that has been reported to exert beneficial effects on human health, including potential therapeutic and neuroprotective effects, particularly in relation to Alzheimer’s disease [46,47,48].

**Figure 1:**
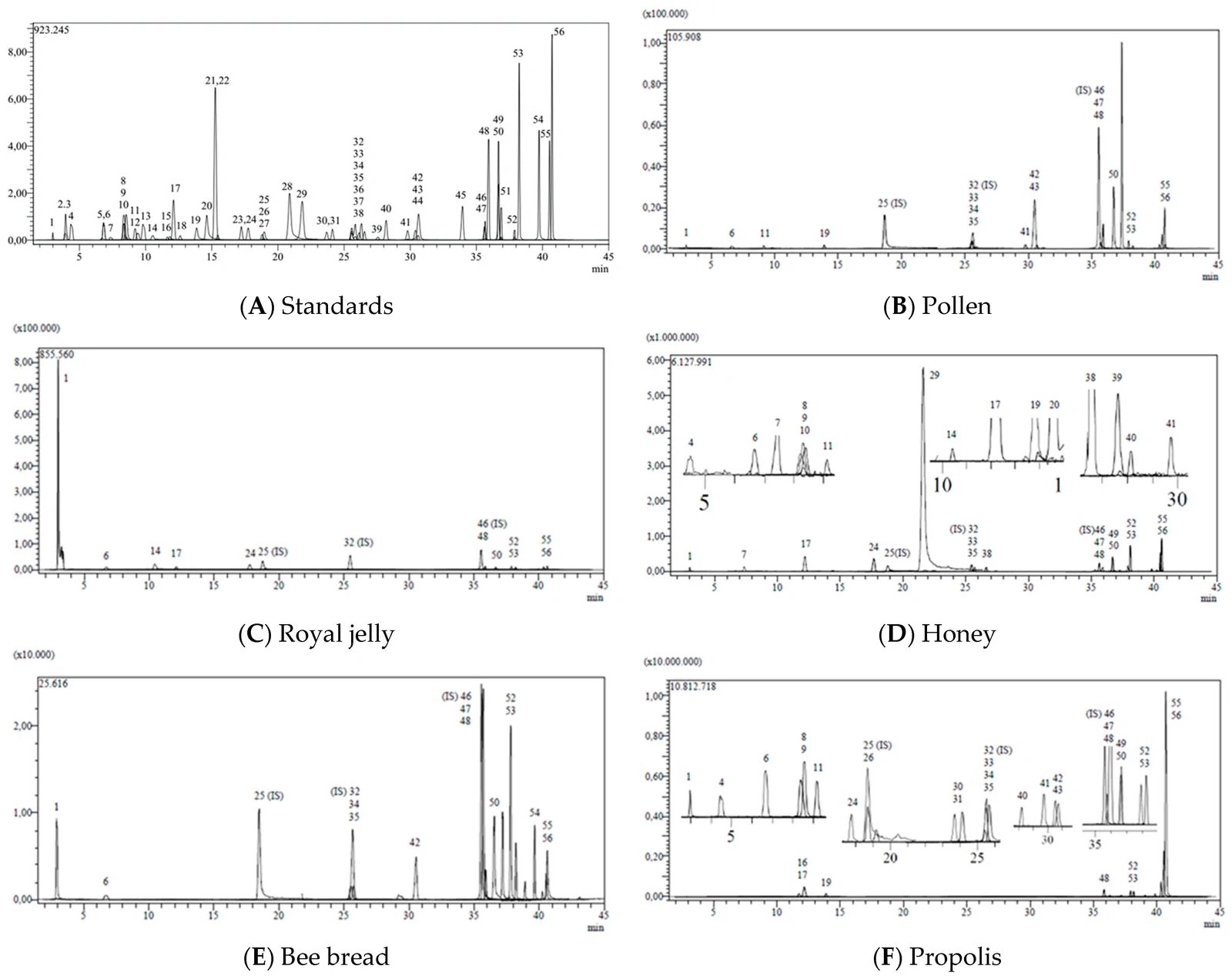
LC-MS/MS chromatogram of samples and standards. (**A**) Standards, (**B**) Pollen, (**C**) Royal jelly, (**D**) Honey, (**E**) Bee bread, (**F**) Propolis.

Quinic acid, which was also detected in the investigated bee products, has been reported to possess several biological activities, including antioxidant, anticancer, anti-inflammatory, antiviral, anti-hepatitis, and antidiabetic effects [49,50]. Considering the diverse biological properties reported for these compounds, their presence and relatively high concentrations in Mersin bee products may contribute, at least in part, to the biological activities associated with these natural products. However, the presence of individual phytochemicals should not be interpreted as direct evidence of a corresponding biological effect, since such activities depend on compound interactions, bioavailability, concentration, and the overall chemical matrix of the bee product. The chromatograms of the bee products and corresponding analytical standards are presented in Figure 1.

### 2.2. Antioxidant Activity Results

The antioxidant capacities of the Mersin bee products were evaluated using DPPH^•^, ABTS^•+^ and DMPD^•+^ radical scavenging assays, and the results were expressed as IC_50_ values (Table 2). Lower IC_50_ values indicate stronger radical scavenging activity. Among the bee products, propolis exhibited the strongest overall antioxidant activity, with IC_50_ values of 33.416 ± 1.253, 27.574 ± 2.545 and 20.163 ± 1.102 for DPPH^•^, ABTS^•+^ and DMPD^•+^, respectively. These results demonstrate that propolis was considerably more effective than the other bee products in neutralizing the tested radicals. The relatively low IC_50_ values of propolis may be associated with its high content of phenolic acids and flavonoids, which can efficiently donate hydrogen atoms or electrons to reactive radicals.

In the DPPH^•^ assay, propolis showed the lowest IC_50_ value among the tested bee products, followed by pollen (71.580 ± 4.122), bee bread (80.889 ± 4.521), royal jelly (88.251 ± 3.594), and honey (101.152 ± 5.682). Under the experimental conditions applied, the IC_50_ values followed the order propolis > pollen > bee bread > royal jelly > honey in terms of radical scavenging activity. The IC_50_ value of propolis was relatively closer to those of the synthetic antioxidant standards BHA (27.413 ± 1.003), α-tocopherol (26.667 ± 1.021), and Trolox (28.295 ± 2.001), providing a useful reference for its antioxidant potential. However, because different extraction procedures were used for the different bee products, these comparisons should be interpreted cautiously.

A similar trend was observed in the ABTS^•+^ assay, although the differences among the bee products were less pronounced. Propolis exhibited the lowest IC_50_ value (27.574 ± 2.545), followed by pollen (32.625 ± 2.121), royal jelly (33.276 ± 1.921), bee bread (41.158 ± 3.476), and honey (107.128 ± 5.683). The higher IC_50_ value observed for honey indicates lower ABTS^•+^ scavenging activity under the applied experimental conditions. The activity of propolis was relatively close to that of BHA (20.724 ± 1.002), α-tocopherol (24.463 ± 1.254), and Trolox (29.787 ± 2.013), suggesting comparatively strong radical scavenging potential. Nevertheless, these comparisons should not be interpreted as evidence of equivalent activity to pure antioxidant compounds.

The DMPD^•+^ assay revealed a somewhat different activity pattern. Propolis again showed the lowest IC_50_ value (20.163 ± 1.102), followed by royal jelly (22.308 ± 1.052), pollen (43.682 ± 3.251), bee bread (62.176 ± 4.589), and honey (65.286 ± 4.356). Trolox exhibited the lowest IC_50_ value among the standards (10.657 ± 0.952), while the IC_50_ values of propolis and royal jelly were relatively close to this reference antioxidant. These results suggest that the measured radical scavenging potential of bee products may vary depending on the chemical characteristics and reaction mechanisms of the radical system employed.

The differences observed among the DPPH^•^, ABTS^•+^, and DMPD^•+^ assays may be related to differences in radical structure, reaction mechanisms, polarity, and the ability of individual compounds to participate in electron- or hydrogen-transfer reactions. Therefore, the use of multiple radical scavenging assays provides complementary information regarding the antioxidant potential of the tested samples. The relatively high coefficients of determination (r^2^ = 0.9206–0.9988) obtained for the samples indicate good linearity of the concentration–response relationships and support the reliability of the calculated IC_50_ values.

In general, propolis exhibited the lowest IC_50_ values among the tested Mersin bee products in all three radical scavenging assays under the experimental conditions used. This observation may be related to the presence and diversity of phenolic compounds and flavonoids commonly reported in propolis. Honey showed comparatively higher IC_50_ values, particularly in the ABTS^•+^ assay. However, since the bee products were subjected to different extraction procedures according to their distinct matrix characteristics, the observed differences should not be considered as a direct quantitative comparison of their intrinsic antioxidant capacities. The results provide preliminary evidence of the antioxidant potential of the extracts, which should be further investigated using standardized extraction procedures and complementary biological studies.

The reducing capacities of the Mersin bee products were evaluated using Fe^3+^, Cu^2+^, and Fe^3+^-TPTZ reduction assays (Table 3). Higher absorbance values indicate greater reducing activity under the respective experimental conditions. Among the tested bee products, propolis showed the highest reducing capacity in all three assays, with absorbance values of 1.207 ± 0.001 at 700 nm, 1.896 ± 0.139 at 450 nm, and 1.381 ± 0.017 at 593 nm. These results indicate a comparatively higher electron-donating potential of the propolis extract under the applied assay conditions. However, considering the different extraction procedures used for the bee products, these findings should be interpreted as assay-specific observations rather than definitive evidence that propolis has intrinsically greater antioxidant capacity than the other bee products.

Pollen and royal jelly exhibited moderately reducing activities, whereas bee bread showed relatively lower values. Honey displayed the lowest reducing capacity in all assays, with values of 0.087 ± 0.004, 0.084 ± 0.007, and 0.269 ± 0.008, respectively. The comparatively higher reducing activity observed for propolis may be associated with its phenolic acid and flavonoid composition, which may contribute to electron-donating capacity.

The results showed generally consistent patterns across the three assays, although the relative responses varied depending on the reducing system. Among the standards, BHA, α-tocopherol, and Trolox generally exhibited higher reducing capacities than the bee product extracts, whereas BHT showed a relatively lower response in the Fe^3+^-TPTZ assay. The high r^2^ values (0.9382–0.9997) indicate good linearity of the concentration–response relationships. Overall, propolis exhibited the highest reducing activity under the applied experimental conditions. However, considering the different extraction procedures used for the bee products, these differences should be interpreted cautiously and should not be regarded as definitive evidence of inherently greater antioxidant capacity.

The total phenolic and flavonoid contents of the Mersin bee products are presented in Table 4. Propolis showed the highest total phenolic content (76.471 ± 4.017 mg GAE/g) and total flavonoid content (15.529 ± 0.543 mg QE/g), followed by royal jelly, pollen, bee bread, and honey. Royal jelly and pollen exhibited similar phenolic and flavonoid levels, whereas honey had the lowest values (36.471 ± 1.992 mg GAE/g and 7.294 ± 0.605 mg QE/g, respectively).

The relatively higher phenolic and flavonoid contents of propolis were accompanied by comparatively higher antioxidant activity in both radical scavenging and reducing-power assays under the applied experimental conditions. Phenolic compounds and flavonoids are recognized as important contributors to antioxidant activity due to their ability to donate electrons or hydrogen atoms and stabilize free radicals. Collectively, these findings suggest that differences in antioxidant responses among the Mersin bee products may be partly associated with differences in their phenolic and flavonoid contents. However, because different extraction procedures were used for the different bee products, these observations should be interpreted cautiously and should not be considered as evidence that propolis is intrinsically the richest or most potent antioxidant source among the tested products.

### 2.3. Enzyme Inhibition Results

The inhibitory effects of Mersin bee products against hCA I, hCA II, AChE, BChE, α-glycosidase and α-amylase are presented in Table 5. Lower IC_50_ values indicate stronger enzyme inhibition. Among the investigated bee products, royal jelly exhibited the strongest inhibitory effect against all tested enzymes, with IC_50_ values of 1.10, 1.90, 1.50, 1.10, 2.15 and 1.40 μg/mL for hCA I, hCA II, AChE, BChE, α-glycosidase and α-amylase, respectively.

Royal jelly showed particularly strong inhibition of hCA I and BChE, with IC_50_ values of 1.10 μg/mL, which were lower than those of the reference inhibitors acetazolamide (5.20 μg/mL for hCA I) and tacrine (2.25 μg/mL for BChE). Similarly, its inhibitory effects against hCA II, AChE, α-glycosidase, and α-amylase were higher than that of the corresponding standard inhibitors. These findings suggest that royal jelly contains bioactive constituents capable of interacting effectively with these enzyme systems.

Among the other bee products, honey and bee bread generally exhibited stronger inhibitory effects than pollen and propolis for several of the tested enzymes. Honey showed IC_50_ values of 7.40 and 6.95 μg/mL against hCA I and hCA II, respectively, while bee bread exhibited values of 8.10 and 8.95 μg/mL. Propolis showed relatively higher IC_50_ values for carbonic anhydrases (19.90 and 22.60 μg/mL), although it demonstrated considerable inhibitory effect against α-glycosidase and α-amylase, with IC_50_ values of 22.17 and 15.90 μg/mL, respectively.

For the cholinesterases, bee products also showed measurable inhibitory effect. Royal jelly displayed the strongest activity, followed by pollen, honey, bee bread, and propolis. The inhibitory effect against AChE and BChE indicate that Mersin bee products may contain compounds with potential relevance to cholinergic enzyme modulation. Likewise, the inhibition of α-glycosidase and α-amylase suggests potential antidiabetic properties, particularly for royal jelly and honey.

The strong enzyme inhibitory effect observed for royal jelly may be related to its diverse composition of phenolic acids, flavonoids, peptides, and other bioactive constituents. Importantly, the high inhibitory effect of royal jelly is consistent with its considerable phenolic and flavonoid contents and antioxidant potential. Collectively, these findings demonstrate that Mersin bee products, particularly royal jelly, possess notable inhibitory potential against carbonic anhydrases, cholinesterases and carbohydrate-hydrolyzing enzymes, supporting their potential as natural sources of bioactive compounds.

### 2.4. Physicochemical Analysis Results of Honey Sample

The physicochemical properties, quality parameters, naphthalene content, and sugar profile of Mersin honey are presented in Table 6.

The proline content was 505.00 ± 8.91 mg/Kg, considerably higher than the Turkish Food Codex Honey Communiqué (TFCHC) minimum limit of 300 mg/Kg, indicating a high-quality honey with respect to this parameter. The diastase activity was also high (25.6 ± 0.173), exceeding the minimum required value of 8. HMF was not detected, remaining well below the maximum limit of 40 mg/Kg and indicating the absence of significant heat-related deterioration.

The moisture content (15.4 ± 0.2%) and electrical conductivity (0.2195 ± 0.063 mS/cm) were below the respective maximum limits of 20% and 0.8 mS/cm. Free acidity was 32.67 ± 1.95 meq/Kg, which was also within the permitted limit of 50 meq/Kg. The pH value was 3.85, consistent with the naturally acidic character of honey. Furthermore, naphthalene was not detected, indicating compliance with the specified maximum limit of 10 µg/Kg.

Regarding the sugar profile, fructose was the predominant sugar (41.04%), followed by glucose (28.37%) and maltose (2.60%), whereas sucrose was not detected. The fructose + glucose content was 69.41%, exceeding the minimum requirement of 60%. The fructose/glucose ratio was 1.45, slightly above the reported range of 0.9–1.4. This relatively high ratio may be associated with the botanical composition and floral sources of the honey and may also influence its crystallization tendency.

Taken together, the physicochemical and quality parameters indicate that Mersin honey generally complies with the requirements of the Turkish Food Codex Honey Communiqué and can be characterized as a high-quality honey. The absence of HMF and naphthalene, together with its high proline and diastase values, further supports its quality and freshness.

A recent study conducted on five monofloral honeys from the Salah Eddine region of Iraq reported favorable physicochemical characteristics, including reduced sugar contents of 75.8–77.7%, fructose/glucose ratios of 0.7–0.9%, sucrose contents of 2.2–2.9%, HMF levels of 17.23–18.87 mg/Kg, electrical conductivity of 0.39–0.46 mS/cm, and pH values of 4.02–4.31. The Iraqi honeys also exhibited measurable antioxidant activity and contained several phenolic compounds, including *p*-coumaric acid, catechin, and quercetin. These findings demonstrate the considerable variability in the physicochemical and phytochemical characteristics of honey according to geographical origin and botanical source [51]. Similarly, the Mersin honey investigated in the present study showed favorable physicochemical characteristics, including high proline and diastase contents, low moisture and free acidity, and non-detectable HMF levels, with most parameters complying with Turkish Food Codex requirements. The Mersin honey also exhibited measurable antioxidant activity and contained several phenolic compounds. Differences between the two regions may be associated with geographical and botanical origin, climatic conditions, and other environmental factors. However, direct quantitative comparison of antioxidant activities should be made cautiously because extraction and analytical procedures differed between studies.

The physicochemical characteristics of the Mersin honey were generally comparable with those reported for honeys from Iasi County, Romania. Romanian honeys showed HMF values ranging from 3.68 to 8.84 mg/Kg in raw and commercial Tilia honeys [52], whereas HMF was not detected in the Mersin honey analyzed in the present study, indicating a lower degree of heat- or storage-related deterioration. The moisture content of Mersin honey was 15.4%, which is consistent with the generally low moisture values expected for high-quality honey. In the Romanian study, differences in acidity and electrical conductivity were observed between raw and commercial samples, demonstrating the influence of processing and botanical origin on these parameters. Similarly, the physicochemical characteristics of Mersin honey may reflect its specific botanical and geographical origin. In general, the non-detectable HMF and low moisture content observed in Mersin honey indicate favorable quality characteristics compared with the Romanian honeys.

A study of four floral honeys from the Fez-Meknes region of Morocco reported total phenolic contents ranging from 22.1 ± 0.4 to 69.3 ± 0.17 mg GAE/100 g and flavonoid contents of 3.6 ± 0.2–7.2 ± 0.6 mg QE/100 g. The multifloral honey also showed notable antioxidant activity, with a DPPH IC_50_ of 30.43 mg/mL and a FRAP IC_50_ of 16.06 mg/mL, and the authors reported negative correlations between phenolic content and antioxidant parameters [53]. In comparison, the Mersin honey investigated in the present study showed measurable antioxidant activity and contained several phenolic compounds identified by LC–MS/MS. Its physicochemical characteristics were also favorable, including a proline content of 505.00 ± 8.91 mg/kg, diastase activity of 25.60 ± 0.173, moisture content of 15.4%, and non-detectable HMF. These findings are broadly consistent with the reported relationship between phenolic constituents and antioxidant activity in honey.

## 3. Materials and Methods

### 3.1. Chemicals

All standards and reagents used for the HS–GC–MS, LC–MS/MS, and HPLC-RID/DAD analyses were obtained from Sigma-Aldrich (Steinheim, Germany). Similarly, the chemicals and reference standards employed in the antioxidant and enzyme inhibition assays were purchased from Sigma-Aldrich Chemie GmbH (Steinheim, Germany). Human carbonic anhydrase isoenzymes I and II (hCA I and hCA II) were purified from human erythrocytes using a Sepharose-4B-L-Tyrosine-sulfanilamide affinity chromatography procedure [54]. The remaining enzymes used in the enzymatic assays were obtained from Sigma-Aldrich (Steinheim, Germany). Unless otherwise specified, all chemicals and reagents were of analytical grade and used without further purification.

### 3.2. Supply of Bee Products

**Figure 2:**
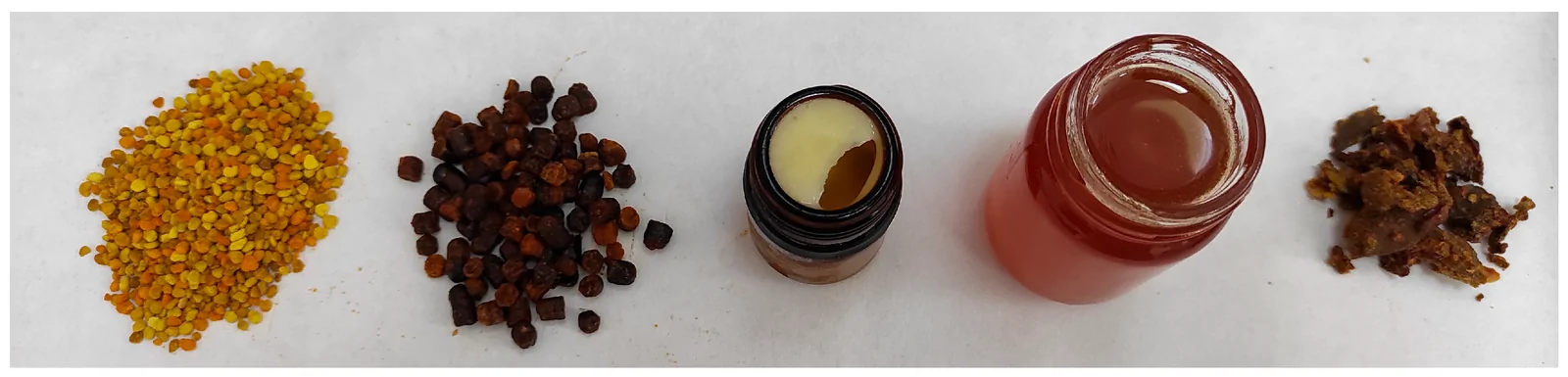
Pollen, bee bread, royal jelly, honey, and propolis samples collected from Mersin Province.

Pollen, bee bread, royal jelly, honey and propolis samples were collected from local beekeepers affiliated with the Mersin Beekeepers’ Association in Mersin Province, Türkiye, a region characterized by active and diverse apicultural production. The honey, pollen, bee bread (perga), and propolis samples analyzed in this study were of polyfloral botanical origin, reflecting the diverse floral resources available in the respective geographical areas. The samples were obtained directly from beekeepers to ensure their traceability and representativeness of locally produced bee products. Due to the high thermal sensitivity and susceptibility to degradation of royal jelly, these samples were collected and transported under cold-chain conditions until further analysis. All collected bee products were appropriately stored prior to laboratory analyses. Representative images of the investigated bee products are presented in Figure 2.

### 3.3. Preparation and Extraction of the Bee Product Extracts

#### 3.3.1. Extraction of Pollen, Propolis and Bee Bread

Prior to extraction, the bee products were subjected to appropriate pretreatment procedures. Due to the waxy and highly viscous nature of propolis at room temperature, the samples were first frozen at −80 °C to facilitate mechanical pulverization. The frozen propolis was subsequently finely powdered using a laboratory blender (Witeg/HG-15D, Langenselbold, Germany). Pollen and bee bread were dried under dark conditions to minimize potential degradation of light-sensitive compounds and were then pulverized to obtain a homogeneous powder.

For the extraction procedure, 10 g of each powdered bee product was accurately weighed and transferred into an appropriate extraction vessel. Each sample was mixed with 50 mL of 70% (*v*/*v*) ethanol, and the mixtures were continuously agitated on a shaker for 48 h to ensure efficient extraction of the target compounds. Following extraction, the mixtures were thoroughly homogenized and subjected to filtration twice through Whatman filter paper to remove residual solid particles. The resulting filtrates were concentrated under reduced pressure using a rotary evaporator (BUCHI/R300, Flawil, Switzerland) until complete removal of the extraction solvent was achieved. The concentrated residues were collected as the corresponding bee product extracts and stored at −20 °C until further analyses. This extraction method was chosen because it is considered one of the most efficient extraction methods in literature.

#### 3.3.2. Extraction of Royal Jelly

For the preparation of the royal jelly extract, 3.0 g of fresh royal jelly was accurately weighed and transferred into a suitable extraction tube. Subsequently, 25 mL of methanol was added to the sample, and the mixture was subjected to continuous agitation on a shaker for 24 h to facilitate the extraction of methanol-soluble constituents. Following the extraction period, the mixture was centrifuged to separate the insoluble protein fraction from the solvent phase. The resulting precipitated proteins were carefully removed, and the clear supernatant was collected for subsequent analysis. The obtained supernatant was used directly for analytical measurements. This extraction method was chosen to precipitate the high protein content present in royal jelly and to better isolate the secondary metabolites [54]. Royal jelly was extracted with methanol because its high protein content may interfere with spectrophotometric analyses. Therefore, the extraction procedure was designed to precipitate and remove proteins as much as possible, thereby minimizing potential matrix interference and facilitating the determination of phenolic and other secondary metabolites.

#### 3.3.3. Extraction of Honey

Briefly, 5 g of honey was transferred into a 50 mL centrifuge tube and diluted to a final volume of 50 mL with ultrapure distilled water, the pH of which had been adjusted to 2.0 using HCl. Prior to sample loading, the SPE (solid-phase extraction) cartridge was conditioned with 10 mL of a 1:1 (*v*/*v*) methanol-distilled water mixture. The resulting 50 mL honey solution was then passed through the SPE cartridge. Subsequently, the cartridge was washed with 20 mL of ultrapure distilled water adjusted to pH 2.0 to remove interfering matrix components. Finally, the retained phenolic compounds were eluted from the SPE cartridge with 2 mL of methanol. The eluates were collected in vials and directly subjected to LC-MS/MS analysis [54]. The main reason for extracting honey using this method was to remove its high carbohydrate content and prevent these components from interfering with or masking the detection of secondary metabolites. The procedure was designed to retain the carbohydrate components on the sorbent and remove them from the sample, thereby facilitating the analysis of secondary metabolites.

### 3.4. Chemical Contents by LC-MS/MS

To determine the secondary metabolites of bee products by LC-MS/MS, the method established by Yilmaz [55] by method validation was applied. Before the application of this method, stock solutions of bee product extracts were prepared at a concentration of 10 mg/mL. These solutions were filtered through a 0.22 μm syringe tip filter and transferred to LC-MS vials before being given to the device. As secondary metabolites, 53 different components were screened and quantitatively analyzed, and three different components were used as internal standards.

The LC-MS/MS analytical parameters are as follows: LC-MS/MS analyses were performed using a Shimadzu LCMS-8040 system (Shimadzu Corporation, Kyoto, Japan), equipped with an electrospray ionization (ESI) source. The system comprised a SIL-30AC autosampler, CTO-10ASvp column oven, LC-30AD dual pumps, and DGU-20A3R degasser (Shimadzu Corporation, Kyoto, Japan), and was controlled using LabSolutions software ver. 5.53 (Shimadzu). Chromatographic separation was achieved using either an Agilent Poroshell 120 EC-C18 column (150 × 2.1 mm, 2.7 µm; Agilent Technologies, Santa Clara, CA, USA) or an Inertsil ODS-4 RP-C18 column (100 × 2.1 mm, 2 µm; GL Sciences Inc., Tokyo, Japan). Acetonitrile and methanol were used as organic mobile phases, with formic acid, acetic acid, ammonium acetate, and ammonium formate employed as mobile-phase additives. The mobile phases consisted of eluent A (water containing 0.1% formic acid and 5 mM ammonium formate) and eluent B (methanol containing 0.1% formic acid and 5 mM ammonium formate). Gradient elution was performed as follows: 20–100% B over 0–25 min, 100% B over 25–35 min, followed by re-equilibration to 20% B from 35 to 45 min. The flow rate was maintained at 0.5 mL/min, the injection volume was 5 µL, and column temperatures of 25, 30, 35, or 40 °C were used depending on the analytical conditions. The mass spectrometer was operated with nitrogen as both nebulizing and drying gas at flow rates of 3 and 15 L/min, respectively. The desolvation line (DL), interface, and heat block temperatures were maintained at 250, 350, and 400 °C, respectively.

### 3.5. Total Phenolic and Flavonoid Content

The total phenolic content of the bee product extracts was evaluated by the Folin–Ciocalteu (F–C) assay following the procedure described by Singleton et al. [56], with minor modifications. Gallic acid was selected as the calibration standard, and the results were reported as gallic acid equivalents (GAE). For calibration, a gallic acid stock solution (10 mg/mL) was prepared by dissolving 10 mg of gallic acid in 10 mL of distilled water. Different volumes of this stock solution (100–1000 µL) were transferred into individual tubes, and distilled water was added to obtain a final volume of 23 mL. Folin–Ciocalteu reagent (0.5 mL) was then added to each solution. After allowing the reaction to proceed for 3 min, 1.5 mL of 2% Na_2_CO_3_ solution was added, followed by thorough mixing. The reaction mixtures were kept at room temperature for 2 h to allow color development. Absorbance values were subsequently recorded at 760 nm using distilled water as the blank. The calibration curve obtained from the gallic acid standards was used to determine the phenolic content of the samples, which was expressed as mg GAE based on the corresponding sample/extract amount.

The total flavonoid content (TFC) of the bee product extracts was determined using the aluminum nitrate colorimetric method described by Park et al. [57], with quercetin serving as the reference standard. For calibration, standard solutions containing 10, 20, 30, 40, and 50 µg of quercetin were prepared from the stock solution. Appropriate volumes of each standard were transferred into separate tubes, followed by the addition of 0.1 mL of 1.0 M potassium acetate (CH_3_COOK) and 0.1 mL of 10% aluminum nitrate [Al(NO_3_)_3_]. Subsequently, 4.3 mL of ethanol was added to each tube, and the mixtures were thoroughly mixed. The reaction solutions were incubated at room temperature for 40 min to allow color development. Absorbance was then measured at 415 nm against a blank prepared using ethanol. The total flavonoid content of the bee product extracts was calculated from the calibration curve obtained with quercetin standards and expressed as micrograms of quercetin equivalents (mg QE).

### 3.6. Antioxidant Activity Assay

#### 3.6.1. Cu^2+^ Reduction Assay -CUPRAC (Cupric Ion Reducing Antioxidant Capacity)

The antioxidant capacity of the bee product extracts was assessed using a modified version of the method proposed by Apak et al. [58]. Briefly, aliquots of the samples at predetermined concentrations were transferred into test tubes and thoroughly mixed using a vortex mixer (VWR International, Radnor, PA, USA). The reaction mixtures were then incubated at room temperature in the dark for 30 min to allow the reaction to proceed. Following incubation, the absorbance values were recorded at 450 nm using a spectrophotometer (Thermo Fisher Scientific, Waltham, MA, USA). Distilled water was used as the blank throughout the assay. For the CUPRAC assay, the antioxidant activity was expressed as absorbance at 450 nm (A_450_), with higher absorbance values indicating greater Cu^2+^-reducing capacity.

#### 3.6.2. Fe^3+^ Reduction Assay -FRP (Ferric Reducing Power)

The reducing power of the bee product extracts was evaluated according to the method originally described by Oyaizu [59], with modifications based on İzol [16]. Briefly, appropriate volumes of the test samples were transferred into test tubes at the predetermined concentrations and thoroughly homogenized using a vortex mixer. Subsequently, 1 mL of distilled water was added to each tube, and the mixtures were vortexed again to ensure complete mixing. The reaction mixtures were then incubated in the dark at room temperature for 10 min. Following incubation, the absorbance of each sample was recorded at 700 nm using a spectrophotometer. All measurements were performed in triplicate to ensure analytical reproducibility. Distilled water was used as the blank, while the control reaction was prepared under identical conditions by replacing the sample solution with an equivalent volume of distilled water. For the Fe^3+^ reducing power assay, the antioxidant activity was expressed as absorbance at 700 nm (A_700_), with higher absorbance values indicating greater reducing power.

#### 3.6.3. FRAP (Fe^3+^-TPTZ Reduction) Assay

The ferric reducing antioxidant power (FRAP) of the bee product extracts was determined using a modified procedure based on the method reported by Bursal et al. [60]. The FRAP reagent was freshly prepared using FeCl_3_ (20 mM, 2.25 mL) and TPTZ (10 mM, 2.25 mL) in acetate buffer (0.3 M, pH 3.6), with the resulting reagent adjusted to a final volume of 2.5 mL. Bee product extracts were prepared in the buffer at concentrations ranging from 15 to 60 µg/mL (this concentration is the sample concentration, not the calculated result concentration). Subsequently, 2.5 mL of each sample solution was mixed with 2.5 mL of FRAP reagent and incubated at 37 °C for 30 min in the dark. The reduction of Fe^3+^–TPTZ to the corresponding Fe^2+^–TPTZ complex was monitored spectrophotometrically at 593 nm. Each concentration was analyzed in triplicate, and the mean absorbance values were used to evaluate the ferric reducing capacity of the samples. For the FRAP assay, the antioxidant activity was expressed as absorbance at 593 nm (A_593_), with higher absorbance values indicating greater Fe^3+^-reducing capacity.

#### 3.6.4. ABTS^•+^ Scavenging Assay

The ABTS radical cation scavenging activity of bee product extracts was determined using a modified version of the method described by Re et al. [61], following the procedure reported by Taslimi et al. [62]. Prior to analysis, the absorbance of the control solution containing the ABTS^•+^ solution and phosphate buffer was adjusted to 1.00 ± 0.025 at 734 nm. Sample solutions were prepared at concentrations ranging from 5 to 20 µg/mL. Appropriate volumes of the sample solutions, 0.1 M phosphate buffer, and 2 mM ABTS^•+^ solution were transferred into test tubes and thoroughly mixed using a vortex mixer. The reaction mixtures were subsequently incubated at room temperature in the dark for 30 min. After incubation, the absorbance values were recorded at 734 nm. Phosphate buffer was used as the blank, and the reduction in absorbance was used to determine the ABTS^•+^ scavenging capacity of the samples. For the ABTS^•+^ scavenging assay, the results were expressed as percentage inhibition and IC_50_ values (µg/mL). Lower IC_50_ values indicated stronger ABTS^•+^ scavenging activity.

#### 3.6.5. DPPH· Scavenging Assay

The DPPH radical scavenging activity of the bee product extracts was determined according to the method of Blois [63], with modifications as described by İzol [16]. A 1 mM solution of 2,2-diphenyl-1-picrylhydrazyl (DPPH) was prepared in ethanol and used as the radical source. Prior to the assay, the absorbance of the control solution, consisting of the DPPH radical solution and ethanol, was adjusted to 1.00 ± 0.025 at 517 nm. Bee product extracts were prepared at concentrations ranging from 15 to 60 µg/mL and transferred into test tubes. This concentration is the sample concentration, not the calculated result concentration. The samples were thoroughly mixed using a vortex mixer and incubated at room temperature in the dark for 30 min. Following incubation, the absorbance of each reaction mixture was measured at 517 nm against an ethanol-containing blank. The decrease in absorbance resulting from the reduction of DPPH radicals was used to evaluate the radical scavenging activity of the samples. For the DPPH radical scavenging assay, the results were expressed as percentage inhibition and IC_50_ values (µg/mL). Lower IC_50_ values indicated stronger DPPH radical scavenging activity.

#### 3.6.6. DMPD^•+^ Scavenging Assay

The DMPD radical cation scavenging activity of the bee product extracts was evaluated according to the method reported by İzol [16]. The DMPD^•+^ reagent was freshly prepared at a final concentration of 0.01 M by combining 1 mL of 0.1 M DMPD solution and 0.2 mL of 0.05 M FeCl_3_ solution with 100 mL of 0.1 M sodium acetate buffer (pH 5.25). Before the assay, the absorbance of the control solution containing the DMPD^•+^ reagent and distilled water was adjusted to 1.00 ± 0.025 at 505 nm. Bee product extracts were prepared at concentrations ranging from 15 to 60 µg/mL (this concentration is the sample concentration, not the calculated result concentration), and appropriate volumes of the samples, distilled water, and DMPD^•+^ reagent were transferred into test tubes. The reaction mixtures were thoroughly homogenized using a vortex mixer and incubated at room temperature in the dark for 50 min. Subsequently, absorbance was measured at 505 nm. Sodium acetate buffer was used as the blank, and the decrease in absorbance was used to assess the DMPD^•+^ scavenging activity of the samples. For the DMPD^•+^ scavenging assay, the results were expressed as percentage inhibition and IC_50_ values (µg/mL). Lower IC_50_ values indicated stronger DMPD^•+^ scavenging activity.

### 3.7. Enzyme Inhibition Assay

#### 3.7.1. Acetylcholinesterase and Butyrylcholinesterase Inhibition Assay

AChE inhibitory effect was determined according to the Ellman method [64]. A 10 mM acetylcholine iodide solution was prepared by dissolving 14.5 mg acetylcholine iodide in 5 mL distilled water. A 1 M Tris-HCl buffer containing EDTA was prepared by dissolving 15.14 g Tris and 0.182 g EDTA in distilled water, adjusting the pH to 8.0, and making the final volume up to 125 mL. A 10 mM DTNB solution was prepared by dissolving 2 mg DTNB and 0.1 g sodium citrate in 10 mL of distilled water. For the assay, 100 µL buffer, 780 µL distilled water, 50 µL DTNB solution, 20 µL AChE enzyme solution, the appropriate volume of sample extract, and 50 µL acetylcholine iodide substrate were added to a final volume of 1 mL. Different concentrations of the extracts were tested. The formation of the yellow-colored 5-thio-2-nitrobenzoic acid product was monitored at 412 nm for 3 min. The percentage of enzyme inhibition was calculated relative to the control, and IC_50_ values were determined from the concentration–enzyme activity curves.

BChE inhibitory effect was determined using the same principle as the AChE assay, with butyrylcholine iodide as the substrate and BChE as the enzyme. A 10 mM DTNB solution was prepared by dissolving 19.8 mg DTNB in 5 mL distilled water. Tris-HCl buffer (1 M) was prepared by dissolving 15.14 g Tris in distilled water, adjusting the pH to 8.0, and making the final volume up to 125 mL. A 5 mM butyrylcholine iodide solution was prepared by dissolving 75.3 mg of the substrate in 50 mL of distilled water. The reaction mixture consisted of 100 µL buffer, 780 µL distilled water, 50 µL DTNB solution, 20 µL BChE enzyme solution, an appropriate volume of sample extract, and 50 µL butyrylcholine iodide substrate, with a final volume of 1 mL. Different extract concentrations were tested, and the absorbance was monitored at 412 nm for 3 min. The percentage inhibition was calculated relative to the control, and IC_50_ values were obtained from the concentration–enzyme activity curves.

#### 3.7.2. α-Amylase Inhibition Assay

The α-amylase inhibition assay was performed according to the method of Xiao et al. [65], with appropriate modifications [16]. A 0.1 M sodium phosphate buffer was prepared by dissolving 5.99 g sodium phosphate in 450 mL distilled water, adjusting the pH to 6.9, and making the final volume up to 500 mL. The starch substrate solution was prepared by dissolving 20 mg starch in 20 mL distilled water with heating and stirring until completely dissolved. The reaction mixture contained 500 µL starch solution, 250 µL α-amylase enzyme solution, 250 µL sodium phosphate buffer, and an appropriate volume of sample extract, with the volume of buffer adjusted accordingly. Different concentrations of the extracts were tested. Absorbance was measured at 580 nm, and the IC_50_ values were calculated from the concentration–enzyme activity curves.

#### 3.7.3. α-Glycosidase Inhibition Assay

The α-glycosidase inhibition assay was performed according to Tao et al. [66], with appropriate modifications. A 0.1 M sodium phosphate buffer was prepared by dissolving 6 g NaH_2_PO_4_ in 450 mL distilled water, adjusting the pH to 6.9, and making the final volume up to 500 mL. A 5 mM p-nitrophenyl-α-D-glucopyranoside (p-NPG) solution was prepared by dissolving 37.6 mg p-NPG in 25 mL of the prepared sodium phosphate buffer. The reaction mixture consisted of 100 µL buffer, 30 µL α-glycosidase enzyme solution, 50 µL p-NPG substrate, an appropriate volume of sample extract, and distilled water to a final volume of 1 mL. Six different concentrations of each bee product extract were tested. Absorbance was monitored at 405 nm for 3 min, and IC_50_ values were calculated from the concentration–enzyme activity curves.

#### 3.7.4. Purification of hCA I and hCA II Isoenzymes

Human CA I and II isoenzymes were purified from human erythrocytes using affinity chromatography according to Çoban et al. [67], with modifications described by İzol [16]. Briefly, 10 mL of erythrocytes was lysed with 40 mL of ice-cold distilled water for 5–10 min and centrifuged at 13,000× *g* for 30 min at 4 °C. The clear hemolysate was collected, adjusted to pH 8.7 with Tris, and stored at 4 °C until purification.

For preparation of the affinity matrix, CNBr-activated Sepharose 4B was coupled with L-Tyrosine and subsequently modified with diazotized sulfanilamide. The prepared gel was thoroughly washed, equilibrated with Tris-HCl buffer (pH 7.8), and packed into a 1 × 10 cm column. The hemolysate was applied to the column, and unbound proteins were removed using 25 mM Tris-HCl/22 mM Na_2_SO_4_ buffer (pH 8.7). CA I was eluted with 1 M NaCl/25 mM Na_2_HPO_4_ buffer (pH 6.3), followed by elution of CA II with 0.1 M NaCH_3_COO/0.5 M NaClO_4_ buffer (pH 5.6). Collected fractions were monitored at 280 nm, and the fractions containing the respective CA isoenzymes were used for subsequent analyses [54].

#### 3.7.5. Carbonic Anhydrase Isoenzymes and Inhibition Assay

The esterase activity of carbonic anhydrase (CA) was determined using p-nitrophenyl acetate (PNPA) as the substrate, based on its hydrolysis to p-nitrophenol/p-nitrophenolate, which exhibits maximum absorbance at 348 nm [68,69]. Since PNPA is sensitive to light and undergoes rapid degradation, the substrate solution was freshly prepared daily, protected from light by aluminum foil, and dissolved in acetone, which minimizes spontaneous hydrolysis.

For the activity assay, Tris-SO_4_ buffer, distilled water, PNPA solution, inhibitor, and CA enzyme solution were combined in a 1 mL cuvette at predetermined concentrations. The blank contained 400 µL buffer, 240 µL distilled water, and 360 µL PNPA solution. Absorbance changes were monitored at 348 nm at 30-s intervals for 3 min against the blank. To evaluate the inhibitory effect of the bee product extracts, six different extract concentrations prepared from DMSO stock solutions were tested under the same conditions. The percentage of residual enzyme activity was plotted against extract concentration, and the IC_50_ values were calculated from the resulting regression equations.

### 3.8. Physicochemical Analysis Methods Applied in Honey

#### 3.8.1. Naphthalene Analysis Method

Naphthalene residues in honey samples were determined using the HS-GC-MS method developed by İzol [25]. Briefly, 1 g of honey was accurately weighed into a 20 mL headspace vial, followed by the addition of naphthalene-D_8_ as an internal standard at a concentration of 10 mg/L in methanol. The sealed vials were placed in the headspace autosampler for instrumental analysis. Analyses were performed using a PerkinElmer Clarus 690 gas chromatograph coupled to a Clarus SQ 8T mass spectrometer and equipped with a TurbaMatrix 40 Trap headspace autosampler (PerkinElmer, Shelton, CT, USA). Chromatographic separation was achieved using an Elite-WAX capillary column (30 m × 0.25 mm i.d., 0.25 µm film thickness; PerkinElmer, Shelton, CT, USA).

The total analysis time was 10 min, with a solvent delay of 3 min and MS data acquisition beginning at 3.5 min and continuing for 10 min. The mass range was set to *m*/*z* 35–250. Instrument operation and data processing were performed using TurbaMass software (Ver. 6.1.2), and compound identification was confirmed by comparison with the NIST spectral library integrated into the instrument software. The calibration curve showed excellent linearity (R^2^ = 0.9959), while the limit of detection (LOD) was 0.5 µg/kg.

#### 3.8.2. Sugar Composition Analysis Method

The sugar composition of honey samples was determined according to the procedures specified by the Turkish Standards Institution (TS 13359) and the International Honey Commission (IHC) as reported by İzol et al. [54]. Briefly, 5 g of honey was accurately weighed and dissolved in approximately 40 mL of ultrapure water. Subsequently, 25 mL of methanol was added, and the mixture was transferred to a volumetric flask. The final volume was adjusted to 100 mL with ultrapure water. The prepared solution was passed through a sterile 0.45 µm syringe filter, and approximately 1.5 mL of the filtrate was transferred into HPLC vials for analysis. Sugar concentrations were quantified as percentages using calibration curves generated from the corresponding standard solutions and their peak areas.

HPLC analysis was performed using ultrapure water and acetonitrile (20:80, *v*/*v*) as the mobile phase at a flow rate of 1.3 mL/min. The injection volume was 10 µL, and the column temperature was maintained at 30 °C.

#### 3.8.3. HMF Analysis Method

The 5-hydroxymethylfurfural (HMF) content of honey samples was determined by high-performance liquid chromatography (HPLC) according to the method of İzol [16], in accordance with the International Honey Commission (IHC) and TS 13356 standards. Briefly, 5 g of honey was accurately weighed into a 50 mL volumetric flask and dissolved thoroughly in 25 mL of ultrapure water. Subsequently, 0.5 mL of Carrez I solution and 0.5 mL of Carrez II solution were added sequentially with thorough mixing after each addition. The volume was then adjusted to 50 mL with ultrapure water, and the mixture was allowed to stand for 3 h. The clarified solution was filtered, and approximately 1.5 mL of the filtrate was further passed through a 0.22 µm membrane filter and transferred into HPLC vials for analysis. HMF concentrations were quantified by comparing the peak areas of the samples with those obtained from the HMF standard.

Chromatographic analysis was performed using a diode-array detector (DAD; Shimadzu Corporation, Kyoto, Japan) and a C18 column (4.6 × 250 mm) maintained at 30 °C. The mobile phase consisted of methanol and ultrapure water (10:90, *v*/*v*), delivered at a flow rate of 1.0 mL/min. The injection volume was 20 µL.

#### 3.8.4. Proline Analysis Method

The proline content of honey samples was determined according to the method described by İzol [16] in accordance with the International Honey Commission (IHC) and TS 13357. Briefly, 5 g of honey was accurately weighed into a 100 mL volumetric flask, dissolved in 50 mL of distilled water, and diluted to the final volume with distilled water. Subsequently, 0.5 mL of the prepared honey solution was transferred into a test tube. For the blank and standard, 0.5 mL of distilled water and 0.5 mL of proline standard solution were used, respectively. Ninhydrin solution (1 mL) and formic acid (1 mL) were added to each tube and mixed for 15 min. The tubes were then heated in a boiling water bath for 15 min, followed by incubation at 70 °C for 10 min. After cooling, 5 mL of 50% 2-propanol was added, and the mixtures were allowed to stand at room temperature for 45 min. Absorbance was subsequently measured at 510 nm. The proline concentration of the honey samples was calculated according to the equation provided in the reference method.

#### 3.8.5. Diastase Analysis Method

Diastase activity in honey samples was determined according to the method used by İzol [16], in accordance with the International Honey Commission (IHC). Briefly, 1 g of honey was accurately weighed, dissolved in acetate buffer, and diluted to 100 mL in a volumetric flask. Aliquots of 5 mL of the prepared honey solution were transferred into test tubes, while 5 mL of acetate buffer was used for the blank. Both tubes were pre-incubated at 40 °C for 5 min in a water bath, after which a Phadebas tablet was added to each tube. The mixtures were vortexed for 10 s and returned to the water bath for 30 min. Subsequently, 1 mL of NaOH solution was added, followed by vortex mixing. The samples were then centrifuged at 3600 rpm for 5 min, and the absorbance of the resulting supernatants was measured at 620 nm using a UV–Vis spectrophotometer (Thermo Fisher Scientific, Waltham, MA, USA), with distilled water as the reference.

#### 3.8.6. Moisture Analysis Method

The moisture content of the honey samples was determined using the refractometric method described by Bogdanov et al. [24], in accordance with the International Honey Commission (IHC) and TS 13365. An Abbe refractometer was used for the measurements. Prior to analysis, each honey sample was thoroughly homogenized, and a small amount was placed on the lower prism of the refractometer to ensure complete coverage of the measurement surface. The upper prism was then carefully closed, and the refractive index was read from the scale through the viewing lens. The corresponding moisture content (%) was subsequently determined using the conversion table provided by Bogdanov et al. [24] and AOAC [70].

#### 3.8.7. Electrical Conductivity Analysis Method

The electrical conductivity of the honey samples was determined according to the procedure specified in TS 13366 [16]. The sample amount was calculated based on the previously determined moisture content to obtain a solution containing at least 20 g of dry matter. The calculated amount of honey was dissolved in approximately 70 mL of distilled water in a 100 mL beaker and quantitatively transferred to a 100 mL volumetric flask. The volume was then adjusted to 100 mL with distilled water. Electrical conductivity was measured using a calibrated conductometer (Mettler Toledo, Greifensee, Switzerland), with the measurement temperature maintained at 20 °C.

#### 3.8.8. Free Acidity Analysis Method

The free acidity of the honey samples was determined according to the procedure specified in TS 13360, following the method described by Bogdanov et al. [24]. Briefly, 10 g of honey was accurately weighed into a 250 mL beaker and dissolved in 75 mL of ultrapure water with continuous stirring. Subsequently, five drops of phenolphthalein indicator were added, and the solution was titrated with 0.05 N NaOH until the endpoint of pH 8.3 was reached, indicated by the appearance of a persistent pale-pink color. The free acidity of each honey sample was calculated using the equation specified in the reference method.

#### 3.8.9. pH Analysis Method

The pH of the honey samples was determined according to the procedure used by İzol et al. [54]. Briefly, 10 g of honey was accurately weighed into a 100 mL beaker and dissolved thoroughly in 75 mL of ultrapure distilled water. Prior to measurement, the pH meter was calibrated using standard buffer solutions at pH 4.00, 7.00, and 10.00. The electrode was then immersed directly into the prepared honey solution, and the pH value was recorded after stabilization of the reading.

### 3.9. Statistical Analysis

All antioxidant activity assays were performed in triplicate, and the results were expressed as mean ± standard deviation (SD). Statistical analyses were conducted using SPSS software (IBM SPSS Statistics for Windows, Version 29.0 (IBM Corp., Armonk, NY, USA)), with Microsoft Excel used for data processing and graphical evaluation. Differences among groups were assessed using one-way analysis of variance (ANOVA), followed by Tukey’s post hoc multiple comparison test. Differences were considered statistically significant at *p* < 0.05.

## 4. Conclusions

The present study provides comparative information on the nutritional, phytochemical, antioxidant, and enzyme inhibitory properties of Mersin bee products under the applied experimental conditions. Propolis generally showed comparatively higher antioxidant responses and total phenolic and flavonoid contents, whereas royal jelly exhibited relatively pronounced inhibitory effect against hCA I, hCA II, AChE, BChE, α-glycosidase, and α-amylase. These findings suggest that the different bee products may possess distinct phytochemical compositions and in vitro bioactivity profiles.

Pollen also showed notable antioxidant and enzyme inhibitory effect together with considerable phenolic and flavonoid contents. However, comparisons among bee products should be interpreted cautiously because different extraction procedures were applied according to the specific physicochemical characteristics of each matrix. Therefore, the observed differences cannot be considered definitive evidence of differences in their intrinsic biological potency.

Mersin honey showed favorable physicochemical characteristics, including relatively high proline and diastase values, low moisture and free acidity, and non-detectable HMF and naphthalene levels. Most of the evaluated quality parameters were within the relevant Turkish Food Codex requirements, indicating satisfactory quality of the investigated honey samples.

In summary, the results provide preliminary evidence of the phytochemical composition and in vitro antioxidant and enzyme inhibitory potential of the investigated Mersin bee products. However, these chemical and in vitro assay results alone cannot establish therapeutic, functional food, or nutraceutical effects. Further studies using standardized extraction procedures, mass-based normalization, detailed characterization of individual bioactive compounds, and appropriate in vivo and clinical models are required to confirm the biological relevance and potential applications of these bee products.

## Figures and Tables

**Table 1:** Phytochemical content results of Mersin bee product extracts (pollen, honey, propolis, bee bread, and royal jelly) (mg analyte/g extract).

No	Secondary Metabolites	R.T.	Pollen	Honey	Propolis	Bee Bread	Royal Jelly
1	Quinic acid	3.0	0.14	0.431	0.185	1.193	19.242
2	Fumaric aid	3.9	-	-	-	-	-
3	Aconitic acid	4.0	-	-	-	-	-
4	Gallic acid	4.4	-	0.038	0.024	-	-
5	Epigallocatechin	6.7	-	-	-	-	-
6	Protocatechuic acid	6.8	0.006	0.438	0.146	0.017	0.111
7	Catechin	7.4	-	1.957	-	-	-
8	Gentisic acid	8.3	-	0.093	-	-	-
9	Chlorogenic acid	8.4	-	0.064	0.176	-	-
10	Protocatechuic aldehyde	8.5	-	0.071	0.422	-	-
11	Tannic acid	9.2	0.01	0.037	0.013	-	-
12	Epigallocatechin gallate	9.4	-	-	-	-	-
13	Cynarine	9.8	-	-	-	-	-
14	*p*-Benzoic acid	10.5	-	0.78	-	-	4.863
15	Epicatechin	11.6	-	-	-	-	-
16	Vanillic acid	11.8	-	-	2.054	-	-
17	Caffeic acid	12.1	-	2.87	3.095	-	0.077
18	Syringic acid	12.6	-	-	-	-	-
19	Vanillin	13.9	0.07	0.841	0.253	-	-
20	Syringic aldehyde	14.6	-	0.216	-	-	-
21	Daidzein	15.2	-	-	-	-	-
22	Epicatechin gallate	15.5	-	-	-	-	-
23	Piceid	17.2	-	-	-	-	-
24	*p*-Coumaric acid	17.8	-	1.466	3.194	-	0.749
25	Ferulic acid-D3-IS	18.8	NA	NA	NA	NA	NA
26	Ferulic acid	18.8	-	-	4.594	-	-
27	Sinapic acid	18.9	-	-	-	-	-
28	Coumarin	20.9	-	0.214	-	-	-
29	Salicylic acid	21.8	-	43.132	-	-	-
30	Cynaroside	23.7	-	-	0.013	-	-
31	Miquelianin	24.1	-	-	0.025	-	-
32	Rutin-D3-IS	25.5	NA	NA	NA	NA	NA
33	Rutin	25.6	0.078	0.049	0.182	-	-
34	Isoquercitrin	25.6	0.352	-	0.325	0.357	-
35	Hesperidin	25.8	0.11	0.261	0.262	0.037	-
36	*o*-Coumaric acid	26.1	-	-	-	-	-
37	Genistin	26.3	-	-	-	-	-
38	Rosmarinic acid	26.6	-	1.396	-	-	-
39	Ellagic acid	27.6	-	0.331	-	-	-
40	Cosmosiin	28.2	-	0.012	0.067	-	-
41	Quercitrin	29.8	0.035	0.043	0.653	-	-
42	Astragalin	30.4	1.129	-	0.472	0.22	-
43	Nicotiflorin	30.6	0.079	-	0.409	-	-
44	Fisetin	30.6	-	-	-	-	-
45	Daidzein	34.0	-	-	-	-	-
46	Quercetin-D3-IS	35.6	NA	NA	NA	NA	NA
47	Quercetin	35.7	0.077	3.068	1.854	0.693	-
48	Naringenin	35.9	0.141	2.769	8.244	0.053	0.018
49	Hesperetin	36.7	-	0.339	0.627	0.059	-
50	Luteolin	36.7	0.082	1.041	1.412	0.06	0.002
51	Genistein	36.9	-	-	-	-	-
52	Kaempferol	37.9	0.075	1.741	3.121	0.137	0.02
53	Apigenin	38.2	0.003	1.876	3.254	0.006	0.003
54	Amentoflavone	39.7	0.005	-	-	0.021	-
55	Chrysin	40.5	0.021	1.98	7.491	0.006	0.02
56	Acacetin	40.7	0.068	5.751	59.381	0.039	0.071

N.: Numbers, -: Not detected, NA: Not applicable, R.T.: Retention time. IS: Internal standard, D3: Deuterium isotope 3.

**Table 2:** Half-maximal inhibition concentration (IC_50_) of Mersin bee products against DPPH, ABTS and DMPD radicals.

Samples		DPPH^·^ Scavenging		ABTS^·+^ Scavenging		DMPD^·+^ Scavenging	
		IC_50_	r^2^	IC_50_	r^2^	IC_50_	r^2^
Standards	BHA	27.413 ± 1.003	0.9547	20.724 ± 1.002	0.9804	40.120 ± 1.085	0.9936
	BHT	59.957 ± 3.011	0.9982	32.110 ± 1.958	0.9938	-	-
	α-Tocopherol	26.667 ± 1.021	0.9507	24.463 ± 1.254	0.9754	-	-
	Trolox	28.295 ± 2.001	0.9758	29.787 ± 2.013	0.9675	10.657 ± 0.952	0.9777
Bee Products	Pollen	71.580 ± 4.122	0.9350	32.625 ± 2.121	0.9484	43.682 ± 3.251	0.9718
	Bee bread	80.889 ± 4.521	0.9761	41.158 ± 3.476	0.9258	62.176 ± 4.589	0.9786
	Propolis	33.416 ± 1.253	0.9988	27.574 ± 2.545	0.9239	20.163 ± 1.102	0.9735
	Honey	101.152 ± 5.682	0.9380	107.128 ± 5.683	0.9897	65.286 ± 4.356	0.9409
	Royal jelly	88.251 ± 3.594	0.9439	33.276 ± 1.921	0.9206	22.308 ± 1.052	0.9813

All values are the mean of three parallel measurements (*n* = 3; the term “triplicates” refers to three technical replicates of the same sample/extract, not three independent biological samples) and are presented as mean ± SD (*p* < 0.05 is considered significant). The IC_50_ concentration is given in μg/mL.

**Table 3:** Fe^3+^, Cu^2+^ and FRAP reduction results of Mersin bee products.

Samples		Fe^3+^ Reduction		Cu^2+^ Reduction		Fe^3+^-TPTZ Reduction	
		λ_700_	r^2^	λ_450_	r^2^	λ_593_	r^2^
Standards	BHA	2.773 ± 0.002	0.9858	1.840 ± 0.022	0.9946	1.250 ± 0.016	0.9905
	BHT	0.379 ± 0.031	0.9896	1.020 ± 0.007	0.9919	0.945 ± 0.034	0.9958
	α-Tocopherol	2.645 ± 0.077	0.9997	2.151 ± 0.019	0.9950	1.755 ± 0.023	0.9932
	Trolox	2.900 ± 0.085	0.9934	1.469 ± 0.016	0.9904	1.181 ± 0.006	0.9912
Bee products	Pollen	0.251 ± 0.002	0.9913	0.294 ± 0.009	0.9869	0.475 ± 0.007	0.9714
	Bee bread	0.160 ± 0.001	0.9820	0.195 ± 0.020	0.9934	0.337 ± 0.003	0.9968
	Propolis	1.207 ± 0.001	0.9993	1.896 ± 0.139	0.9957	1.381 ± 0.017	0.9934
	Honey	0.087 ± 0.004	0.9479	0.084 ± 0.007	0.9549	0.269 ± 0.008	0.9382
	Royal jelly	0.263 ± 0.003	0.9951	0.204 ± 0.006	0.9945	0.547 ± 0.007	0.9689

All values are the mean of three parallel measurements (*n* = 3, the term “triplicates” refers to three technical replicates of the same sample/extract, not three independent biological samples) and are presented as mean ± SD (*p* < 0.05 is considered significant). Concentrations are given in µg/mL.

**Table 4:** Results of total phenolic and flavonoid content of Mersin bee products.

Bee Products	Total Phenolic (mg GAE/g Dry Extract)	Total Flavonoid (mg QE/g Dry Extract)
Pollen	41.176 ± 2.012	8.235 ± 0.601
Propolis	76.471 ± 4.017	15.529 ± 0.543
Bee bread	38.824 ± 1.785	7.765 ± 0.512
Honey	36.471 ± 1.992	7.294 ± 0.605
Royal jelly	41.765 ± 1.412	8.353 ± 0.508

The values given are the three parallel measurements’ means ± SD. GAE: Gallic acid equivalents, QE: Quercetin equivalents. The term “triplicates” refers to three technical replicates of the same sample/extract, not three independent biological samples.

**Table 5:** hCA I, hCA II, AChE, BChE, α-glycosidase and α-amylase enzyme inhibition results of Mersin bee products.

Bee Products	hCA I		hCA II		AChE		BChE		α-Glycosidase		α-Amylase	
	IC_50_	r^2^	IC_50_	r^2^	IC_50_	r^2^	IC_50_	r^2^	IC_50_	r^2^	IC_50_	r^2^
Pollen	10.10	0.988	9.35	0.9779	8.50	10.10	9.12	0.9768	9.75	0.9766	-	-
Bee bread	8.10	0.9589	8.95	0.9678	5.25	8.10	4.54	0.9961	9.26	0.9884	10.55	0.9778
Propolis	19.90	0.9865	22.60	0.9766	20.55	19.90	17.53	0.9921	22.17	0.9666	15.90	0.9776
Honey	7.40	0.9974	6.95	0.9581	5.25	7.40	6.30	0.9889	8.51	0.9871	6.37	0.9866
Royal Jelly	1.10	0.9577	1.90	0.9828	1.50	1.10	2.15	0.9912	1.40	0.9588	1.90	0.9914
Acetazolamide	5.20	0.9895	3.75	0.9887	-	-	-	-	-	-	-	-
Tacrine	-	-	-	-	2.25	0.9883	2.25	0.9883	-	-	-	-
Acarbose	-	-	-	-	-	-	-	-	5.85	0.9890	5.85	0.9890

Acetazolamide served as the reference inhibitor for hCA I and hCA II, while tacrine and acarbose were employed as reference inhibitors for AChE/BChE and α-glycosidase/α-amylase, respectively. Concentrations are expressed in μg/mL.

**Table 6:** Proline, diastase, HMF, moisture, electrical conductivity, free acidity, pH, naphthalene and sugar results of Mersin honey.

Analyzes	Honey	Limit Value of TFCHC	Methods
Proline (mg/Kg)	505.00 ± 8.91	300 (min)	TS 13357 [16]
Diastase	25.6 ± 0.173	8 (min)	IHC (5.1) [16]
HMF (mg/Kg)	N.D.	40 (max)	TS 13356 [16]
Moisture (%)	15.4 ± 0.2	20 (max)	TS 13365 [16]
Electrical conductivity (mS/cm)	0.2195 ± 0.063	0.8 (max)	TS 13366 [16]
Free acidity (meq/Kg)	32.67 ± 1.95	50 (max)	TS 13360 [16]
pH	3.85		TS 13360 [16]
Naphthalene (µg/Kg)	N.D.	10 (max)	[25]
Glucose (%)	28.36716		TS 13359 [16]
Fructose (%)	41.04222		TS 13359 [16]
Sucrose (%)	N.D.	5 (max)	TS 13359 [16]
Maltose (%)	2.60421	4 (max)	TS 13359 [16]
Fructose + Glucose	69.4093	60 (min)	TS 13359 [16]
Fructose/Glucose	1.44682	0.9–1.4 (%)	TS 13359 [16]

N.D.: Not determined. TS: Turkish standard.

## Data Availability

The original contributions presented in this study are included in the article.
